# Inhibition of α-Synuclein Aggregation and Mature Fibril Disassembling With a Minimalistic Compound, ZPDm

**DOI:** 10.3389/fbioe.2020.588947

**Published:** 2020-10-16

**Authors:** Samuel Peña-Díaz, Jordi Pujols, Francisca Pinheiro, Jaime Santos, Irantzu Pallarés, Susanna Navarro, María Conde-Gimenez, Jesús García, Xavier Salvatella, Esther Dalfó, Javier Sancho, Salvador Ventura

**Affiliations:** ^1^Institut de Biotecnologia i Biomedicina, Universitat Autònoma de Barcelona, Barcelona, Spain; ^2^Departament de Bioquímica i Biologia Molecular, Universitat Autònoma de Barcelona, Barcelona, Spain; ^3^Department of Biochemistry and Molecular and Cell Biology, Institute for Biocomputation and Physics of Complex Systems (BIFI), University of Zaragoza, and Aragon Institute for Health Research (IIS Aragon), Zaragoza, Spain; ^4^Institute for Research in Biomedicine (IRB Barcelona), The Barcelona Institute of Science and Technology, Barcelona, Spain; ^5^ICREA, Barcelona, Spain; ^6^Medicine, M2, Universitat Autònoma de Barcelona (UAB), Barcelona, Spain; ^7^Faculty of Medicine, University of Vic-Central University of Catalonia (UVic-UCC), Barcelona, Spain

**Keywords:** α-synuclein, protein aggregation, amyloid inhibitor, Parkinson’s disease, synucleinopathies, small molecules

## Abstract

Synucleinopathies are a group of disorders characterized by the accumulation of α-Synuclein amyloid inclusions in the brain. Preventing α-Synuclein aggregation is challenging because of the disordered nature of the protein and the stochastic nature of fibrillogenesis, but, at the same time, it is a promising approach for therapeutic intervention in these pathologies. A high-throughput screening initiative allowed us to discover ZPDm, the smallest active molecule in a library of more than 14.000 compounds. Although the ZPDm structure is highly related to that of the previously described ZPD-2 aggregation inhibitor, we show here that their mechanisms of action are entirely different. ZPDm inhibits the aggregation of wild-type, A30P, and H50Q α-Synuclein variants *in vitro* and interferes with α-Synuclein seeded aggregation in protein misfolding cyclic amplification assays. However, ZPDm distinctive feature is its strong potency to dismantle preformed α-Synuclein amyloid fibrils. Studies in a *Caenorhabditis elegans* model of Parkinson’s Disease, prove that these *in vitro* properties are translated into a significant reduction in the accumulation of α-Synuclein inclusions in ZPDm treated animals. Together with previous data, the present work illustrates how different chemical groups on top of a common molecular scaffold can result in divergent but complementary anti-amyloid activities.

## Introduction

Parkinson’s disease (PD) is an incurable disorder that affects around 0.3% of the population and more than 1% of people over 60 years of age (4% over 80 years), being the second most prevalent neurodegenerative disease worldwide (Nussbaum and Ellis, 2003; Dexter and Jenner, 2013; Kalia and Lang, 2015). Together with Dementia with Lewy Bodies (DLB) and Multiple System Atrophy (MSA), PD is part of a group of human disorders known as synucleinopathies (Spillantini et al., 1998a,b; Fanciulli and Wenning, 2015). Intracellular proteinaceous inclusions constitute the main culprit of neuronal damage and disease progression in the synucleinopathies, although the aggregates accumulate in different cell types and affect distinct brain regions depending on the disease (Fellner et al., 2011; Luk et al., 2012). These abnormal protein deposits are mostly composed of aggregated α-synuclein (α-Syn). In the particular case of PD, α-Syn aggregation occurs in the dopaminergic neurons of *substantia nigra pars compacta*. As a consequence, PD suffering patients display reduced dopamine levels, which results in the archetypic motor and non-motor symptoms of the disease (Spillantini et al., 1997). Indeed, single-point mutations and multiplications of the gene that encodes for α-Syn (*SNCA*) (Singleton et al., 2003; Ibanez et al., 2004) have been related to familial cases of PD with early-onset (Polymeropoulos et al., 1997), thus reinforcing the connection between α-Syn and PD.

α-Syn is an intrinsically disordered protein highly expressed in the brain and associated with vesicle trafficking in healthy conditions (Bendor et al., 2013). In pathological situations, α-Syn aggregates into oligomers and amyloid fibrils that compromise cellular homeostasis, exert toxicity and ultimately lead to neuronal death (Serpell et al., 2000). Remarkably, diffusible aggregated species can be internalized by healthy neighboring neurons, where they seed the aggregation of soluble α-Syn molecules, a mechanism that has been compared with the templated conformational conversion occurring in prion diseases (Hansen et al., 2011). As it occurs in prions, α-Syn assemblies can present diverse structural arrangements, forming strains (Li et al., 2018) that differ in their aggregation properties (Bousset et al., 2013), and target distinct brain regions and cell types (Lau et al., 2020).

Many of the current therapeutic approaches for PD aim to reduce the neuronal load of aggregated α-Syn, either by targeting the α-Syn polypeptide directly or through indirect approaches such as the stimulation of degradation pathways (Spencer et al., 2009; Decressac et al., 2013; Xilouri et al., 2013) and gene silencing (Faustini et al., 2018; Kantor et al., 2018; Lassot et al., 2018; Zharikov et al., 2019). Among them, the identification of small compounds that might act as chemical chaperones blocking the aggregation and propagation of α-Syn or, in the best-case scenario, dismantling α-Syn mature aggregates into non-toxic species is receiving increasing attention. However, the disordered nature of α-Syn, together with the multiplicity of conformationally different species that populate the aggregation process, imposes significant difficulties for the rational design of effective α-Syn binders. For this reason, high-throughput screening (HTS) of large chemical libraries has become a significant focus of research in the hunt for disease-modifying lead compounds (Silva et al., 2011).

**FIGURE 1:**
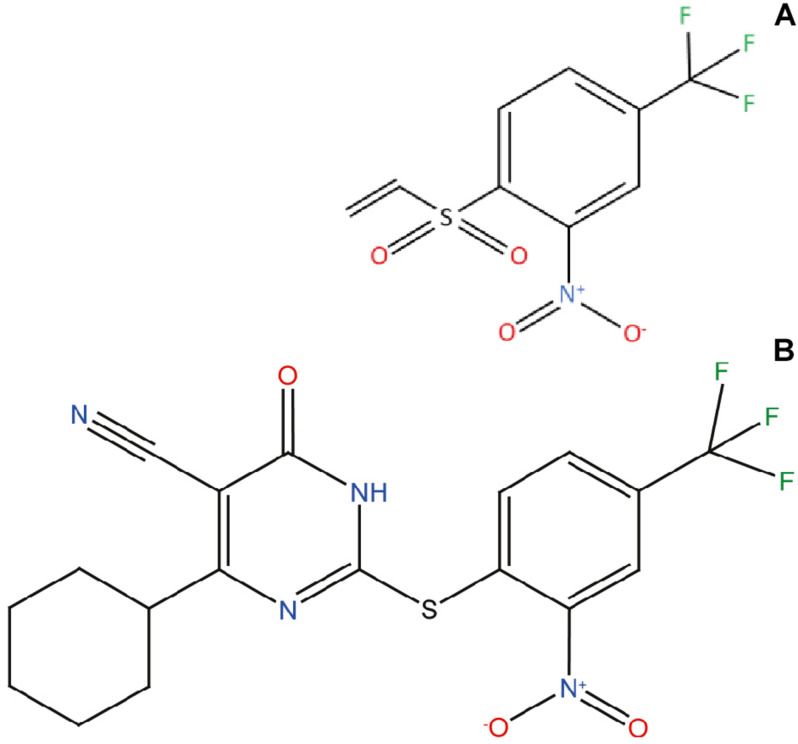
ZPDm, a ZPD-2 minimal structure. Chemical structures of **(A)** 2-nitro-4-(trifluoromethyl)phenyl vinyl sulfone, named ZPDm, and **(B)** ZPD-2, both constituted by a hydrophobic core formed by aromatic rings and polar projections.

To analyze the inhibitory potential of a chemical library with more than 14,000 chemically diverse structures, we optimized a robust HTS screening protocol (Pujols et al., 2017) based on Thioflavin-T (Th-T) fluorescence, light-scattering measurements and Transmission Electron Microscopy (TEM). This pipeline was used to discover and characterize small molecules that act as potent inhibitors of α-Syn amyloid formation, such as SynuClean-D (SC-D) (Pujols et al., 2018) and ZPD-2 (Pena-Diaz et al., 2019). In the present work, we present and characterize ZPDm (Figure 1), a novel molecule identified in the primary HTS screen. ZPDm was the smallest compound in the library displaying a significant inhibitory potency at a substoichiometric concentration. ZPDm prevents the aggregation of *wild type* α-Syn and the familial A30P and H50Q variants. The molecule acts preferentially at the late stages of the polymerization reaction, suggesting that it targets preferentially ordered aggregates. Indeed, ZPDm is highly effective at disaggregating the mature α-Syn fibrils of different strains. To the best of our knowledge, it constitutes the minimal synthetic molecule that *co*njugates inhibitory and α-Syn fibril disrupting activity in the same chemical scaffold. These *in vitro* activities are translated into the ability to reduce significantly α-Syn aggregation in a well-established *Caenorhabditis elegans* (*C. elegans*) model of PD.

## Materials and Methods

### Protein Expression and Purification

WT α-Syn and its variants (H50Q and A30P) were expressed and purified as previously described (Pujols et al., 2017); the obtained protein was lyophilised and kept at -80°C until its use.

### *In vitro* Aggregation of α-Syn

Lyophilised α-Syn was carefully resuspended in sterile PBS 1X and filtered through 0.22 μm membrane to discard small aggregates. Aggregation was performed at 70 μM of α-Syn (WT, H50Q or A30P) in a sealed 96-well plate, in a total volume of 150 μL. 40 μM Th-T in PBS 1X, a 1/8” diameter Teflon polyball (*Polysciences Europe GmbH, Eppelheim, Germany*) and 100 μM ZPDm, (trifluoromethyl)benzene or DMSO (in control samples) were also added to each well. The plate was incubated at 37°C and 100 rpm fixed in an orbital shaker Max-Q 4000 (Thermo Fisher Scientific, Waltham, Massachusetts, United States). Every 2 h, Th-T fluorescence was measured in a Victor3.0 Multilabel Reader (PerkinElmer, Waltham, Massachusetts, United States), exciting through a 430–450 nm filter and collecting emission signal with a 480–510 filter. Each assay was done in triplicate and the values of the kinetic fitted according to the following equation:

(1) $$\propto =1-\frac{1}{k_{b}\left(e^{k_{a}t-1}\right)+1}$$

where *k*_*b*_ and *k*_*a*_ constitute the homogeneous nucleation rate constant and the autocatalytic rate constant, respectively (Crespo et al., 2016).

ZPDm was added at different concentrations in the titration assays (200, 150, 100, 75, and 50 μM). In time-dependent assays, 7 independent aggregation reactions were prepared simultaneously and incubated as aforementioned in a 96-well plate as triplicates. 100 μM of ZPDm were added at different time points after the reaction had begun (4, 8, 12, 16, 20, and 24 h). In all cases α-Syn concentration was constant at 70 μM. An equivalent volume of DMSO was added to the control sample at the beginning of the reaction.

For the study of the disaggregation assays, α-Syn 70 μM was incubated in a 96-well plate as previously described for 2 days and Th-T fluorescence measured. Then, ZPDm was added to a final concentration of 100 μM. The plate was incubated for an additional 24 h and Th-T fluorescence was measured.

Strains were generated as previously described (Bousset et al., 2013; Peelaerts et al., 2015; Li et al., 2018; Carija et al., 2019). Briefly, α-Syn was resuspended in PBS 1X and dialysed for 24 h in a 1:1,000 (v/v) ratio with either buffer B (50 mM Tris-HCl pH 7.0) or buffer C (50 mM Tris-HCl pH 7.0, 150 mM NaCl). Dialysed samples were filtered through 0.22 μm membranes and incubated at 70 μM for 2 days as described above. ZPDm was added to a final concentration of 100 μM and plates incubated for additional 24 h. Then, Th-T fluorescence was measured.

### Light-Scattering

80 μL of end-point aggregates were collected, placed into a quartz cuvette and analyzed in a Cary Eclipse Fluorescence Spectrophotometer (Agilent, Santa Clara, CA, United States) by exciting at 300 and 340 nm and 90^*o*^ collecting between 280 and 360 nm.

### Transmission Electron Microscopy (TEM)

End-point α-Syn aggregates were collected and Diluted 1/10 (v/v) in PBS 1X. Diluted samples were gently sonicated for 5 min and 5 μL of the resultant sample were placed on a carbon-coated copper grid for 5 min. Using a filter paper, the grids were dried to remove the excess of sample and washed twice with miliQ water. Finally, 5 μL of 2% (w/v) uranyl acetate were added and left incubate for 2 min. As previously indicated, the excess of uranyl acetate was removed, and grids were left to air-dry for 10 min. Images were obtained using a Transmission Electron Microscopy Jeol 1400 (Peabody, MA, United States) operating at an accelerating voltage of 120 kV. A minimum of 30 fields were screened per sample, in order to collect representative images.

### Protein Misfolding Cyclic Amplification (PMCA)

PMCA protocol was performed as previously described (Herva et al., 2014). Briefly, α-Syn was resuspended in Conversion Buffer (PBS 1X, 1% Triton X-100, 150 mM NaCl) to a final concentration of 90 μM and supplemented with Complete Protease Inhibitor Mixture (Roche Applied Science, Penzberg, Germany). 60 μL of this solution were loaded into 200 μL PCR tubes containing 1.0 mm silica beads (Biospec Products, Bartlesville, OK, United States). α-Syn was then exposed to 24 h cycles of 30 s sonication and 30 min of incubation at 37°C, using a Misonix 4000 sonicator, at 70% power. The incubated sample was recovered after each 24 h cycle and 1 μL was added to a new PCR tube containing fresh α-Syn at 90 μM. In the case of ZPDm treated samples, the compound was added to the fresh sample in each step to a final concentration of 128 μM, which corresponds to the 0.7:1 α-Syn:ZPDm ratio of the previous aggregation assays. Untreated samples were prepared adding the same concentration of DMSO (0.26%) present in the treated mixtures. This process was repeated for 5 days. All the reactions were made in triplicate.

Ten microliter of aggregated samples at the end of each cycle were diluted 1:10 with 90 μL of PBS 1X, 40 μM Th-T. Th-T fluorescence emission was measured in a Cary Eclipse Fluorescence Spectrophotometer (Agilent, Santa Clara, CA, United States), by exciting the samples at 445 nm and collecting the emission signal between 460 and 600 nm.

### Proteinase K Digestion

18 μL of PMCA-aggregated α-Syn were incubated with 6 μL of Proteinase K (5 μg/mL as final concentration) for 30 min at 37°C. Then, 8 μL of loading buffer containing 1% β-mercaptoethanol was added and the sample was incubated 10 min at 95°C for PK inactivation. Finally, 7 μL of the samples were loaded into a Tricine-SDS-PAGE gel. Unstained Protein Standard markers (Thermo Fisher Scientific, Waltham, MA, United States) were used as a reference. Gels were stained with Blue safe.

### Nuclear Magnetic Resonance (NMR)

^15^N-labeled human WT α-Syn was expressed in *E. coli BL21 DE3* strains. Cells were grown in LB medium until the optical density (OD) at 600 nm reached a level of 0.6. Cultures were then centrifuged at 3,000 rpm for 15 min and the obtained pellets resuspended in 1 L of minimal medium: 768 mL of miliQ water with 1 mL of ampicillin 100 mg/mL, 100 μL CaCl_2_ 1M, 2 mL MgSO_4_ 2 M, 20 mL glucose 20%, 10 mL vitamins 100x (Sigma-Aldrich, Darmstadt, Germany), 200 mL salts M9 and 1 g ^15^NH_4_ (Cambridge Isotope Laboratories, Inc., Tewksbury, MA, United States). Cells were incubated for 1 h at 250 rpm and 37°C. Finally, 1 mM IPTG was added to induce protein expression for 4 h. Protein was purified as previously described (Pujols et al., 2017).

### Caenorhabditis Elegans Assays

#### Maintenance

Animals synchronization was carried out by bleaching and overnight hatching in M9 (3 g/L KH_2_PO_4_, 6 g/L Na_2_HPO_4_, 5 g/L NaCl, 1 M MgSO_4_) buffer. Thus, nematodes were cultured at 20°C on growth media plates (NGM) containing 1 mM CaCl_2_, 1 mM MgSO_4_, 5 μg/mL cholesterol, 250 M KH_2_PO_4_ pH 6.0, 17 g/L Agar, 3 g/L NaCl. Plates were previously seeded with *E. coli OP50* strain. Nematodes were maintained using standard protocols (Brenner, 1974).

#### Strains

Strain NL5901, *unc-119(ed3) III; pkIs2386* [*Punc-54::α-SYN::YFP; unc-119(+)*] was obtained from the *C. elegans* Genetic Center (CGC). For the α-Syn induced dopaminergic degeneration analysis, strain UA196 (Harrington et al., 2012), gifted generously by the laboratory of Dr. Guy Caldwell (Department of Biological Science, The University of Alabama, Tuscaloosa, United States), was used; [*sid-1(pk3321]; baIn33* [*Pdat-1::sid-1, Pmyo-2::mCherry*]; *baIn11* [*Pdat-1::α-SYN; Pdat-1::GFP*]). In the main text, this strain was named *Pdat-1::GFP; Pdat-1::α-SYN*.

#### ZPDm Administration

After cooling, the autoclaved NGM agar medium (1 mM CaCl_2_, 1 mM MgSO_4_, 5 μg/mL cholesterol, 250 M KH_2_PO_4_ pH 6.0, 17 g/L Agar, 3 g/L NaCl) was enriched with 100 μM of a stock solution of 4 mM ZPDm in 0.2% DMSO to a final concentration of 10 μM. After 2 days, plates were seeded with 250 μL of *E. coli OP50* with 10 μM of ZPDm. Nematodes were placed on the plates at larval stages L4 and exposed either to ZPDm or DMSO (controls) for 7 days. Daily transfer was done to avoid cross progeny.

### Aggregate Quantification

The number of cellular inclusions was quantified as previously described (van Ham et al., 2008; Munoz-Lobato et al., 2014). Briefly, NL5901 (*Punc-54::α-SYN::YFP*) worms were age-synchronized and left overnight to hatch. Nematodes in phase L1 were cultured and grown into individual NGM plates seeded with *E. coli* OP50. When animals reached L4 developmental stage, they were transferred onto either ZPDm treated plates or DMSO treated plates (negative control). Every day, animals were transferred into a new plate to avoid cross contamination. At stage L4+7, the aggregates in the anterior part of every single animal were counted. For each experiment, thirty 7-days old nematodes per treatment were analyzed using a Nikon Eclipse E800 epifluorescence microscope equipped with an Endow GFP HYQ filter cube (Chroma Technology Corp., Bellows Falls, Vermont United States) and each experiment was carried out in triplicate. Inclusions could be described as discrete bright structures, with edges distinguishable from surrounding fluorescence. ImageJ software was used for measuring the number of cellular aggregates considering the area dimensions. For the quantification of α-syn aggregates in *C. elegans* one single image was taken from each animal. However, every image contained among 30–45 stacks (1 μm) that allowed to detect aggregates that are at different positions.

### Microscopy and Imaging

Animals were placed in a 1 mM solution of sodium azide and mounted with a coverslip on a 4% agarose pad. Animals were visualized with a Nikon Eclipse E800 epifluorescence microscope. The system acquires a series of frames at specific *Z*-axis position (focal plane) using a *Z*-axis motor device. Animals were examined at 100 × magnification to examine α-Syn induced DA cell death and at 40× to examine α-Syn apparent aggregate.

### Statistical Analysis

All graphs were generated with GraphPad Prism 6.0 software (GraphPad Software Inc., La Jolla, CA, United States). Data were analyzed by two-way ANOVA Tukey test using SPSS software version 20.0 (*IBM Analytics, Armonk, NY, United States*) and *t*-test using GraphPad software version 6.0 (GraphPad Software Inc., La Jolla, CA, United States). All data are shown as means and standard error of mean (SEM). P < 0.05 was considered statistically significant. In the graphs ^∗^, ^∗∗^, and ^∗∗∗^ indicate *p* < 0.05, *p* < 0.01, and *p* < 0.001, respectively.

## Results

### ZPDm Inhibits α-Synuclein Aggregation *in vitro*

ZPDm is a molecule that was initially identified as a positive hit in the HTS performed by our lab back in 2017 on top of the HitFinder^TM^ chemical library from Maybridge (Pujols et al., 2017). Although they were found independently, structurally, ZPDm is a minimalistic version of ZPD-2 (Pena-Diaz et al., 2019) with a reduced MW, LogP, and TPSA, which, in principle, would increase its drug-likeness (Figure 1 and Table 1). Still, ZPDm implements the generic physicochemical properties common to most of the published inhibitors of α-Syn aggregation, namely, a planar hydrophobic core formed by aromatic rings that interact with apolar exposed regions in α-syn assemblies. This core is frequently coated with polar projections that interfere with hydrophobic packing and disrupt intermolecular hydrogen bonds; they difficult elongation and, eventually, might promote fibril disassembly (Pujols et al., 2020). A benzene ring constitutes the hydrophobic moiety of ZPDm, connected to three polar groups—vinyl sulfone, nitro, and trifluoromethyl – in carbon positions 1, 2, and 4, respectively. Remarkably, the ZPD-2 chemical structure displays the same functional groups in carbons 1, 2, and 4 of the primary phenyl ring. However, the vinyl sulfone is substituted by a sulfide group that extends the molecule to incorporate additional aromatic moieties, which we previously assumed to be critical for its activity (Pena-Diaz et al., 2020). Thus, ZPDm can be considered as a building block for the synthesis of the more complex ZPD-2 molecule. Considering these structural differences, we performed a set of orthogonal experiments to contrast if, as ZPD-2, ZPDm might turn to be a lead compound with significant α-Syn anti-aggregational activity.

**TABLE 1:** SwissADME predicted properties of ZPDm and ZPD-2.

	ZPDm	ZPD-2
Molecular weight (g/mol)	281.21	424.4
Heavy atoms	18	29
Aromatic heavy atoms	6	12
H-bond acceptors	7	8
H-bond donors	0	1
TPSA (Å^2^)	88.34	140.66
Log P_*O/W*_	2.42	3.9
Solubility (mg/mL)	9.28E-02	1.94E-03
GI absorption	High	Low
Drug-like (Lipinski)	0 violations	0 violations
Drug-like (Veber)	0 violations	1 violations
Drug-like (Egan)	0 violations	2 violations
Leadlikeness	0 violations	2 violations

**FIGURE 2:**
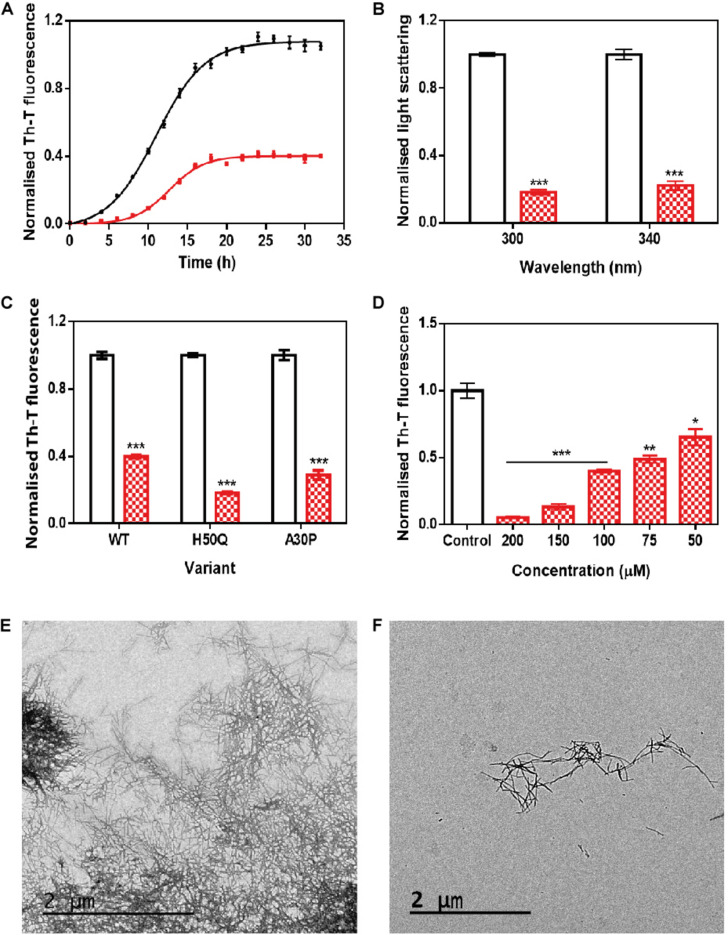
*In vitro* analysis of the capacity of ZPDm to inhibit α-Syn aggregation. **(A)** α-Syn aggregation kinetics in the absence (black) and presence (red) of 100 μM of ZPDm followed by Th-T fluorescence. **(B)** Light-scattering measurements at 300 and 340 nm, in the absence (white) and presence (red) of ZPDm. **(C)** H50Q and A30P α-Syn variants aggregation in the absence (white) and presence (blue) of ZPDm. **(D)** Inhibition of α-Syn aggregation with different concentrations of ZPDm. **(E,F)** Representative TEM images in the absence **(E)** and presence **(F)** of ZPDm. Th-T fluorescence is plotted as normalized means. Final points were obtained at 48 h. Error bars are represented as SE of mean values; ^∗^*p* < 0.05, ^∗∗^*p* < 0.01, and ^∗∗∗^*p* < 0.001. ZPDm prevents the aggregation of WT, A30P, and H50Q α-Syn variants *in vitro*, even at substoichiometric ratios.

The incubation of 70 μM α-Syn with 100 μM ZPDm inhibited the protein aggregation, decreasing the final Th-T fluorescence signal by 60% compared to control untreated samples (Figure 2A). Fitting of the kinetic data to a typical sigmoidal nucleation-polymerization reaction revealed that ZPDm diminishes the primary nucleation rate constant by eightfold (k_*b*_ = 0.0034), relative to the control reaction (k_*b*_ = 0.0275), at the expenses of a slightly higher autocatalytic rate constant, with k_*a*_ of 0.449 and 0.323 h^–1^ for ZPDm treated and untreated samples, respectively. This results in t_0_ and t_1/2_ being increased by 4 and 2 h, respectively, in the compound’s presence. Light-scattering measurements at 300 and 340 nm at the end of the reaction reported a decrease of 81 and 78% in the dispersed light (Figure 2B) in the presence of ZPDm, respectively, consistent with a reduction of the total aggregated material. The visual inspection of α-Syn samples by TEM corroborated a reduction in the number of amyloid fibrils per field in the presence of ZPDm (Figure 2F), compared to untreated samples (Figure 2E). A titration assay in which we incubated 70 μM α-Syn in the presence of decreasing amounts of ZPDm indicated a dose-dependent inhibition, with a statistically significant activity at a substoichiometric concentration of 50 μM, at which ZPDm still reduces the Th-T signal at the end of the reaction by 35% (Figure 2D).

We further examined if ZPDm was able to prevent the aggregation of two mutants of α-Syn, H50Q, and A30P, which have been associated with familial PD (Kruger et al., 1998; Appel-Cresswell et al., 2013). The incubation of these α-Syn variants with ZPDm reduced Th-T fluorescence at the end of the reaction by 81 and 71% for H50Q and A30P, respectively (Figure 2C).

ZPDm, ZPD-2, and SC-D and other positive hits in the library, share a common property, the presence of a trifluoromethyl group connected to an aromatic ring. We hypothesized that perhaps we were in front of the minimal inhibitory unit, which might be very useful for future Structure Activity Relationship (SAR) studies. Therefore, we synthesized the (trifluoromethyl)benzene moiety and assessed its anti-aggregational potential (Supplementary Figure S1). Both kinetic data using Th-T and light scattering measurements converged to indicate that this molecule is devoid of any activity, suggesting that it might be necessary, but not sufficient to endorse ZPDm with the above-described anti-aggregation properties.

### ZPDm Prevents α-Syn Aggregation in Protein Misfolding Cyclic Amplification Assays

**FIGURE 3:**
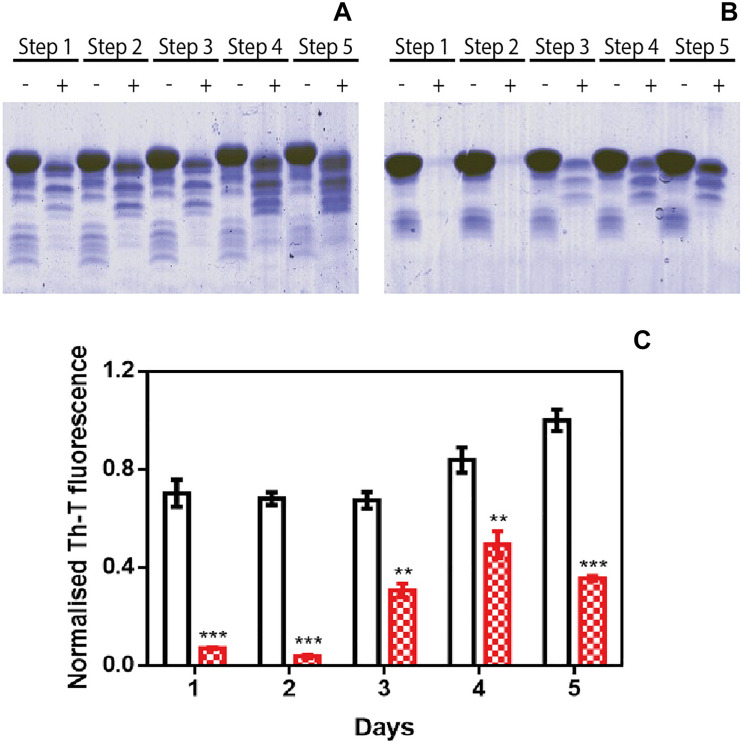
Inhibitory activity of ZPDm in PMCA assays. **(A,B)** Bis/Tris SDS-PAGE gels of PMCA samples in the absence **(A)** and presence **(B)** of ZPDm, before (–) and after (+) PK digestion. **(C)** Th-T fluorescence of different PMCA cycles in samples treated (red) and untreated (white) with ZPDm. Th-T fluorescence is plotted as normalized means. Error bars are represented as SE of mean values; ^∗∗^*p* < 0.01 and ^∗∗∗^*p* < 0.001. ZPDm anti-aggregation activity results in delayed formation of PK-resistant and Th-T positive amyloid structures.

We used protein-misfolding cyclic amplification (PMCA) to test the inhibitory capacity of ZPDm under continuous seeding conditions. Based on the nucleation-dependent polymerization model for prion replication, PMCA is a technique that forces aggregation to happen by seeding soluble α-Syn with preformed fibrils (Jung et al., 2017). After the first round of fibril elongation, aggregates are sonicated and used as seeds for the second cycle of PMCA. A representative sample from each cycle is then treated with proteinase K (PK) and analyzed by SDS-PAGE, to evidence fibril formation, since in contrast to soluble α-Syn, the fibrils are significantly resistant to proteolysis. Using this protocol, PK-resistant species could be observed already in the 1st cycle of PMCA in untreated samples, with a maximum of PK resistance at the 4th cycle (Figure 3A). Th-T fluorescence measurements of the same samples indicated that this protection correlates with the presence of amyloid-like assemblies (Figure 3C). In contrast, in the presence of ZPDm, PK-resistant species are absent until the 3rd cycle, and they never reach the levels of the control samples, although certain adaptation of misfolded α-Syn to ZPDm seems to occur in cycles 4th and 5th (Figure 3B). The Th-T fluorescence signal is negligible in the two first PMCA cycles and significantly lower than that of control samples at any considered cycle (Figure 3C).

### ZPDm Exhibits Amyloid Disaggregation Activity *in vitro*

ZPD-2 does not interact with soluble and monomeric α-Syn and, therefore, is not expected to interfere with the protein’s functional state. Nuclear Magnetic Resonance ^1^H-^15^N HSQC spectra of N^15^ labeled α-Syn in the presence and absence of ZPDm, indicates that this is also the case for this smaller molecule since we could not identify any perturbations in chemical shifts or peak intensities in the spectra (Supplementary Figure S2).

**FIGURE 4:**
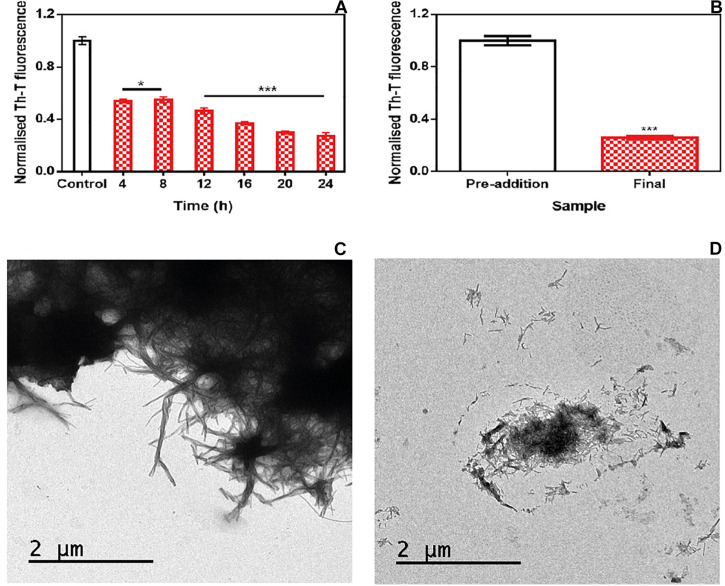
Disaggregational activity of ZPDm. **(A)** Th-T fluorescence of α-Syn end-point aggregates after the addition of ZPDm at different time points during the aggregation kinetics. **(B)** Th-T fluorescence assay before and 24 h after the addition of ZPDm to mature α-Syn fibrils. **(C,D)** Representative TEM images in the absence **(C)** and presence **(D)** of ZPDm. Th-T fluorescence is plotted as normalized means. Error bars are represented as SE of mean values; *p < 0.05 and ***p < 0.001. ZPDm inhibitory capacity increases with the reaction progress, indicating that the compound may interact with aggregated structures and disentangle them.

To address the time window in which ZPDm remains active, we set up an experiment in which a constant amount of ZPDm was added to different aggregation reactions at different time intervals after the reaction has begun (Figure 4A). To our surprise, the respective Th-T signals indicated that the anti-amyloid activity increased as the reaction progressed, which is in stark contrast with the behavior of ZPD-2, which was mostly active when added at the early stages of the reaction and inactive when added at the plateau phase (Pena-Diaz et al., 2019). The time-dependent activity profile of ZPDm can only be explained if this compound recognizes the Th-T positive aggregated species and exerts an intense fibril disruption activity. To confirm this extent, α-Syn mature fibrils were incubated for 24 h with ZPDm. The Th-T fluorescence analysis revealed that treated samples suffered a signal reduction of 74% (Figure 4B). This data was supported by TEM images, which illustrated the disruption of large fibrillar clusters into shorter fibrils or amorphous aggregates (Figures 4C,D).

**FIGURE 5:**
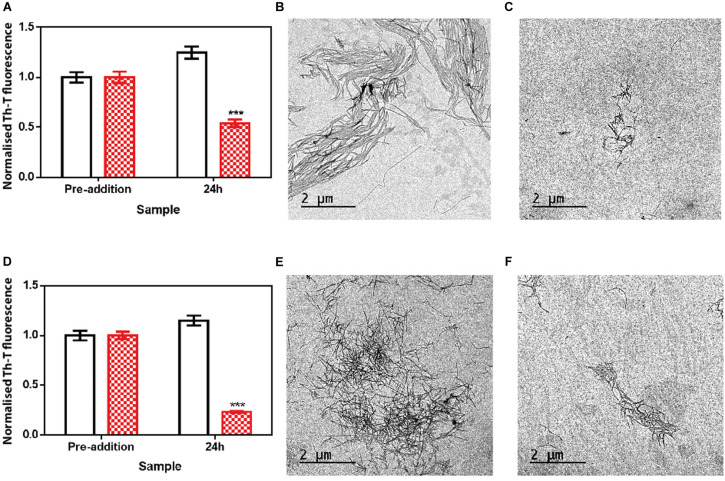
Disaggregational effect of ZPDm in preformed fibrils of two different strains. **(A)** Strain B aggregates disaggregation in the presence (red) and absence (white) of ZPDm as monitored by Th-T fluorescence. **(B,C)** Representative TEM images of untreated **(B)** and ZPDm treated **(C)** samples. **(D)** Strain C aggregates disaggregation in presence (red) and absence (white) of ZPDm as monitored by Th-T fluorescence. **(E,F)** Representative TEM images of untreated **(E)** and ZPDm treated **(F)** samples. Data are shown as means, and error bars are shown as the SE of means; ***p < 0.001. The disaggregational ability of ZPDm is also observed in two morphologically different α-Syn strains.

We assessed if this amyloid-disrupting activity was independent of the conformational properties of the mature fibrils, by aggregating α-Syn in 50 mM Tris–HCl pH 7.0 in the absence or presence of 150 mM NaCl, which generates two different strains, known as strain B and C, respectively (Bousset et al., 2013; Carija et al., 2019). As shown in Figure 4, the addition of ZPDm to the mature fibrils of these strains promoted a significant decrease in the amount of amyloid-like material as monitored both by Th-T fluorescence and TEM 24 h after the addition of the molecule (Figure 5). These data suggest that in contrast to ZPD-2, insensitive to preformed amyloid fibrils, ZPDm is endorsed with a generic and potent disaggregation activity.

### ZPDm Decreases the Formation of α-Syn Aggregates in a *C. elegans* Model of PD

**FIGURE 6:**
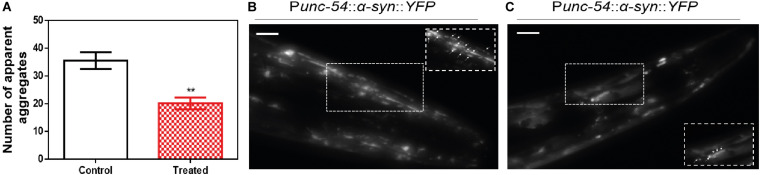
Inhibition of α-Syn inclusions formation in a *C. elegans* model. **(A)** Quantification of α-Syn muscle inclusions per area in NL5901 worms in the absence (white) and presence of ZPDm (red). **(B,C)** Representative images of apparent α-Syn muscle aggregates obtained by epifluorescence microscopy of NL5901 worms treated without **(B)** and with ZPDm **(C)**. (Scale bars, 10 μm). Between 40 and 50 animals were analyzed per condition. Aggregates are indicated by white arrows. Data are shown as means, and error bars are shown as the SE of means; **p < 0.01. NL5901 *C. elegans* strain forms visible accumulations of aggregated α-Syn that are reduced when ZPDm is administered.

We decided to test if the ZPDm *in vitro* activity can be translated *in vivo* to a simple animal model of PD. To do so, we employed the well-described strain NL5901 of *C. elegans.* In this strain, α-Syn is fused to Yellow Fluorescence Protein (YFP) and expressed under the control of *the unc-54* promoter, transgene *pkIs2386 [Punc-54::α-SYN::YFP]* (Hamamichi et al., 2008; van Ham et al., 2008), generating protein inclusions in body wall muscle cells. ZPDm was administered in the food at 10 μM final concentration to animals at the L4 stage, and they were examined 7 days later, 9 days after hatching (L4+7). These aged worms are intended to mimic aged PD patients. We used epifluorescence microscopy to visualize the fluorescent aggregates. The images demonstrated that ZPDm reduced the formation of muscular inclusions by 43% (Figure 6A), with an average of 20.2 ± 2.13 apparent α-Syn aggregates in treated worms (Figure 6C) compared with the 35.2 ± 3.04 observed in control samples (Figure 6B).

## Discussion

The identification of small compounds that may abrogate the process of protein aggregation in neurodegenerative disorders is attracting increasing interest, both in academia and industry (Pujols et al., 2020).

The lack of structural information about the intermediate species that populate the reaction, and the intrinsically disordered nature of many of the proteins behind these diseases, has made it challenging to use of structure-guided drug design for amyloid inhibitors. Only recently, the high-resolution structures of the fibrils formed by proteins connected to different amyloidosis have allowed the rational design of peptides that interfere with the growth or seeding of the fibrils (Seidler et al., 2018; Saelices et al., 2019; Sangwan et al., 2020). However, because peptides usually display poor pharmacokinetics, which should be significantly optimized before they become drugs, small molecules are still the preferred option for the treatment of the diseases caused by the aggregation of proteins within the brain.

The screening of large chemical libraries in the search for α-Syn aggregation inhibitors has provided potent molecules like anle138b (Wagner et al., 2013), BIOD303 (Moree et al., 2015), SynuClean-D (Pujols et al., 2018), 582032 (Toth et al., 2019) or the collection of compounds recently reported by Kurnik et al. (2018). All these molecules display two or more aromatic rings in their structures, a property that is shared by active polyphenols like curcumin (Pandey et al., 2008), EGCG (Bieschke et al., 2010), and baicalein (Jiang et al., 2010), repurposed inhibitors like Fasudil (Tatenhorst et al., 2016), and LMTM (Schwab et al., 2017) or compounds generated by rational design like NPT100-18A (Wrasidlo et al., 2016). Usually, the aromatic rings form a planar hydrophobic core that is thought to interact with apolar exposed regions in α-Syn or its assemblies. However, despite the presence of multiple aromatic groups is recurrent in natural α-Syn aggregation inhibitors and many of the reported screening efforts result in the identification of this kind of molecules, several natural compounds exhibiting a single aromatic ring have been shown to act as α-Syn aggregation modulators (Supplementary Figure S3), including scyllo-inositol, gallic acid, dopamine, safranal and caffeic acid (Herrera et al., 2008; Di Giovanni et al., 2010; Liu et al., 2014; Ibrahim and McLaurin, 2016; Save et al., 2019).

We recently used a robust high-throughput screening pipeline to uncover molecules able to modulate α-Syn fibrillation (Pujols et al., 2017). Among the active compounds, we searched for a small compound bearing a single aromatic ring. We identified ZPDm, which, interestingly enough, is a minimal version of ZPD-2, a potent inhibitor identified in the same library (Pena-Diaz et al., 2019), with half of its heavy aromatic atoms. This opened an opportunity to approach a comparative SAR for these molecules.

ZPDm reduces the *in vitro* aggregation of WT α-Syn and the protein’s A30P and H50Q familial variants in a 60%, or higher, at a 0.7:1 (protein: ZPDm) ratio. This activity was orthogonally confirmed by light-scattering and TEM. Moreover, the inhibitory activity of ZPDm reduced the number of PK-resistant and Th-T positive species in PMCA assays, thus interfering with α-Syn templated seeding and/or aggregates amplification (Herva et al., 2014). It should be explored whether adaptation of α-Syn to ZPDm at late PMCA stages might translate in some resistance to the molecule during aggregates propagation.

Solution NMR measurements indicated that ZPDm was not interacting with soluble α-Syn monomers, and, therefore, it is not expected to impact the physiological function of the protein. Moreover, the addition of ZPDm at different time points of the aggregation reaction suggested that ZPDm is mainly active at the latest stages of the aggregation and indeed, further analysis demonstrated that the molecule is capable of disassembling mature α-Syn amyloid fibrils generated under different solution conditions, conceptually similar to the α-Syn strains observed in different synucleinopathies (Bousset et al., 2013; Peelaerts et al., 2015). Importantly, these features translate into a significant reduction in the number of apparent aggregates in the muscular cell wall of a *C. elegans* model of PD when the compound is added in the food at a concentration of 10 uM; whether this i*n vivo* anti-aggregational effect results in animal phenotypic benefits should be further explored.

The above-described results illustrate how despite ZPD-2 and ZPDm share a significant part of their chemical structure and both are effective α-Syn aggregation inhibitors, their mechanism of action differs significantly, with ZPD-2 acting preferentially at the early stages of the fibrillation and becoming inactive once the polymerization has advanced significantly, being devoid of detectable fibril disrupting activity.

In contrast, ZPDm is more effective at later stages and behaves as a robust disaggregating agent. The fact that (trifluoromethyl)benzene is an inactive molecule indicates that the bulk of the structure shared by ZPD-2 and ZPDm acts as a building block and that the particular chemistry and spatial disposition of the groups that decorate this moiety are responsible for the different mode of action of these compounds. For instance, the (trifluoromethyl)benzene contains the aromatic ring common to the vast majority of small active compounds. However, it lacks a strong hydrogen bond donor/acceptor, which is another characteristic common to many of these molecules (Supplementary Figure S3). Therefore, our data suggest that these are the minimum requirements for an active α-Syn aggregation inhibitor. The aromatic rings would allow interactions with hydrophobic regions, and the polar groups might disrupt the abundant short inter-strand hydrogen bonds that contribute to the amyloid structure’s sustainment. Indeed, despite their different size, the number of hydrogen bonds acceptors in ZPD-2 and ZPDm is fairly similar (Table 1). Despite speculative, the preferential affinity for early-stage aggregates exhibited by ZPD-2 could be explained by its extended aromatic core and higher Log P_*O/W*_, which might facilitate interactions with exposed hydrophobic patches in oligomers and small aggregates. In contrast, the compact structure of ZPDm might allow targeting defined binding pockets at the ends of amyloid fibrils, interfering with fibril elongation, and eventually disrupting pre-formed non-covalent interactions.

From a pharmacokinetic point of view, ZPDm is predicted to be more soluble than ZPD-2, to exhibit a higher gastrointestinal absorption and better drug-likeness (Table 1). ZPDm is also predicted to be a better lead compound from a medicinal chemistry perspective than ZPD-2 (Table 1).

To the best of our knowledge, the only other active molecule with a single aromatic ring derived from the screening of a large chemical library is the compound 576755 (Toth et al., 2019), which in addition to a benzene ring, displays the expected hydrogen bonds acceptors/donors (Supplementary Figure S3). 576755 is a potent aggregation inhibitor that acts at the oligomerization stage both *in vitro* and *in cells*. However, it was identified in a screening for compounds that interact with monomeric α-Syn, and therefore, is not expected to have fibril disrupting activity. Indeed, it did not impact fibril transmission (Toth et al., 2019), consistent with its activity and being complementary to that of ZPDm, a fibril anti-propagating agent in PMCA assays.

Overall, here we describe a new small molecule with the potential to be converted into a lead compound and, perhaps more importantly, together with previous data, envision a way to design minimal aromatic molecules with different α-Syn anti-aggregational activities rationally. Because ZPD-2 and ZPDm function on the same target but have complementary activity, it will be interesting to test if a combination of them can have a synergic effect that overpasses the individual molecules’ potential.

## Data Availability Statement

All datasets presented in this study are included in the article/Supplementary Material.

## Author Contributions

SV designed the research. SP-D, JP, FP, JST, SN, MC-G, JSC, JG, and ED performed the research. SP-D, JP, IP, XS, JSC, ED, and SV analyzed the data. SP-D, JP, and SV wrote the manuscript. All authors contributed to the article and approved the submitted version.

## Conflict of Interest

The authors declare that the research was conducted in the absence of any commercial or financial relationships that could be construed as a potential conflict of interest.

## Supplementary Material

Supplementary Figure 1 Inhibitory capacity of (trifluoromethyl)benzene. **(A)** Chemical structures of (trifluoromethyl)benzene, (left) and ZPDm (right). **(B)** α-Syn aggregation kinetics in the absence (black) and presence (green) of 100 μM of (trifluoromethyl)benzene followed by Th-T fluorescence. **(C)** Light-scattering measurements at 300 and 340 nm, in the absence (white) and presence (green) of (trifluoromethyl)benzene.

Supplementary Figure 2 NMR analysis of ZPDm lack ofinteraction with monomeric α-Syn. ^1^H-^15^N HSQC NMR spectra of 15N-labeled α-Syn (70 μM) in the presence **(A)** and in the absence **(B)** ofZPDm (100 μM). The superposition of the two NMR spectra is shown in **(C)**.

Supplementary Figure 3 Chemical structures of different α-Syn aggregation inhibitors with asingle aromatic ring. Chemical structures of **(A)** scyllo-inositol, **(B)** gallic acid, **(C)** dopamine, **(D)** epinephrine, **(E)** norepinephrine, **(F)** thymoquinone, **(G)** safranal, **(H)** caffeic acid, **(I)** ferulic acid, **(J)** protocathecuic acid, **(K)** tyrosol, and **(L)** 576755.
