# Supplementary figures and images for: Inhibition of α-Synuclein Aggregation and Mature Fibril Disassembling With a Minimalistic Compound, ZPDm
Source: Front Bioeng Biotechnol. 2020 Oct 16;8:588947. doi: 10.3389/fbioe.2020.588947 (PMC7597392; doi:10.3389/fbioe.2020.588947)

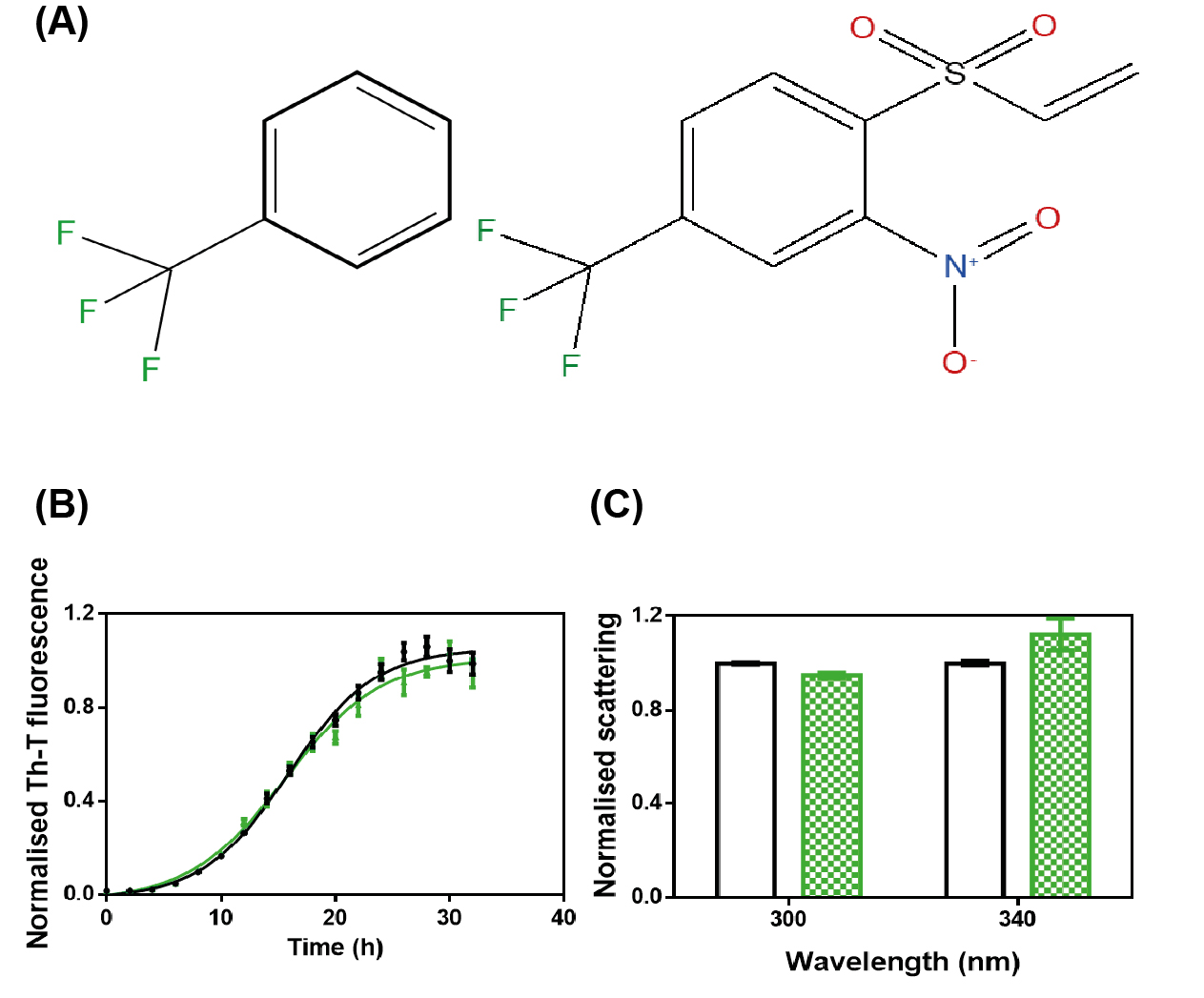

Supplement: Supplementary Figure 1 — Inhibitory capacity of (trifluoromethyl)benzene. (A) Chemical structures of (trifluoromethyl)benzene, (left) and ZPDm (right). (B) α-Syn aggregation kinetics in the absence (black) and presence (green) of 100 μM of (trifluoromethyl)benzene followed by Th-T fluorescence. (C) Light-scattering measurements at 300 and 340 nm, in the absence (white) and presence (green) of (trifluoromethyl)benzene. [file Image_1.JPEG]

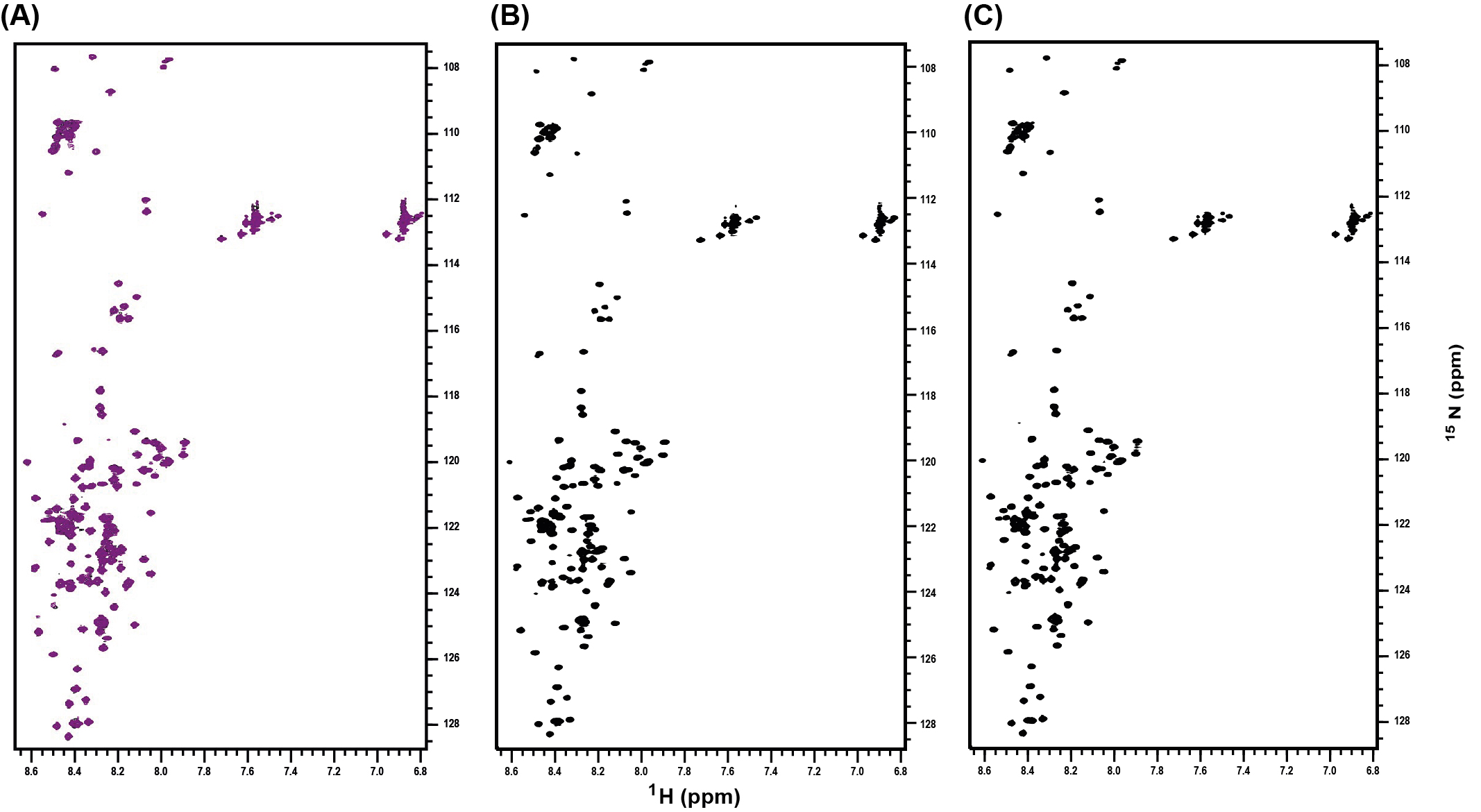

Supplement: Supplementary Figure 2 — NMR analysis of ZPDm lack ofinteraction with monomeric α-Syn. 1H-15N HSQC NMR spectra of 15N-labeled α-Syn (70 μM) in the presence (A) and in the absence (B) ofZPDm (100 μM). The superposition of the two NMR spectra is shown in (C). [file Image_2.JPEG]

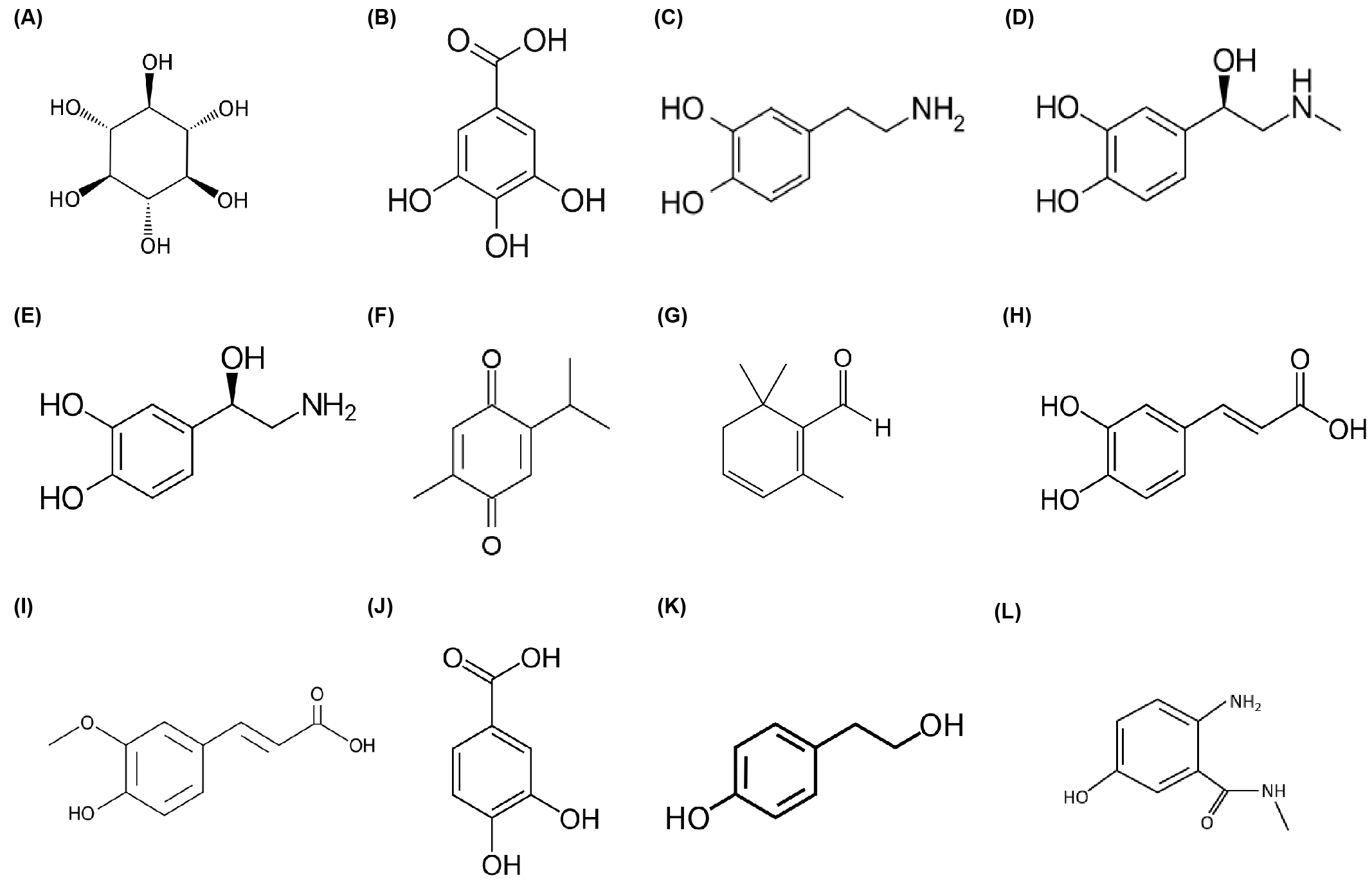

Supplement: Supplementary Figure 3 — Chemical structures of different α-Syn aggregation inhibitors with asingle aromatic ring. Chemical structures of (A) scyllo-inositol, (B) gallic acid, (C) dopamine, (D) epinephrine, (E) norepinephrine, (F) thymoquinone, (G) safranal, (H) caffeic acid, (I) ferulic acid, (J) protocathecuic acid, (K) tyrosol, and (L) 576755. [file Image_3.JPEG]
